# Hunting modulators of plant defence: the grapevine trunk disease fungus *Eutypa lata* secretes an amplifier for plant basal immunity

**DOI:** 10.1093/jxb/eraa152

**Published:** 2020-03-03

**Authors:** Pingyin Guan, Terigele, Florian Schmidt, Michael Riemann, Jochen Fischer, Eckhard Thines, Peter Nick

**Affiliations:** 1 Molecular Cell Biology, Botanical Institute, Karlsruhe Institute of Technology, Fritz-Haber-Weg, Karlsruhe, Germany; 2 Institut für Biotechnologie und Wirkstoff-Forschung gGmbH,, Kaiserslautern, Germany

**Keywords:** Basal immunity, *Eutypa lata*, fungal culture extracts, grapevine trunk diseases, modulators, *O*-methylmellein

## Abstract

Grapevine trunk diseases (GTDs) are progressively affecting vineyard longevity and productivity worldwide. To be able to understand and combat these diseases, we need a different concept of the signals exchanged between the grapevine and fungi than the well-studied pathogen-associated molecular pattern and effector concepts. We screened extracts from fungi associated with GTDs for their association with basal defence responses in suspension cells of grapevine. By activity-guided fractionation of the two selected extracts, *O*-methylmellein was identified as a candidate modulator of grapevine immunity. *O*-Methylmellein could not induce immune responses by itself (i.e. does not act as an elicitor), but could amplify some of the defence responses triggered by the bacterial elicitor flg22, such as the induction level of defence genes and actin remodelling. These findings show that *Eutypa lata*, exemplarily selected as an endophytic fungus linked with GTDs, can secrete compounds that act as amplifiers of basal immunity. Thus, in addition to elicitors that can trigger basal immunity, and effectors that down-modulate antibacterial basal immunity, once it had been activated, *E. lata* seems to secrete a third type of chemical signal that amplifies basal immunity and may play a role in the context of consortia of mutually competing microorganisms.

## Teaser

*O*-Methylmellein is an amplifier of flg22-triggered immunity to grapevine trunk diseases.

## Introduction

In contrast to most animals, plants are sessile organisms, which makes immunity even more crucial in warding off intruders. However, plants lack a somatic adaptive immune system. Instead, they have evolved innate immunity of two layers: pathogen-associated molecular pattern (PAMP)-triggered immunity (PTI), a broad-spectrum basal immunity triggered by molecular patterns generic for a class of microbes (microbe-associated molecular patterns and PAMPs); and effector-triggered immunity (ETI), which is often specific for one pathogen and usually accompanied by programmed cell death (for a recent review see Boutrot and Zipfel, 2017).

The most comprehensive understanding of plant responses to PAMPs has been achieved for the PTI triggered by bacterial flagellin (Gómez-Gómez and Boller, 2002). This building block of bacterial flagella harbours a conserved peptide motif, flg22, which is recognized by the flagellin-sensing 2 (FLS2) receptor on the plasma membrane. Binding of the ligand leads to a conformational change of this transmembrane receptor, which activates a calcium influx channel, an apoplastic oxidative burst, rapid responses of the cytoskeleton, activation of mitogen-activated protein kinase signalling, and activation of a transcriptional cascade involving transcription factors such as WRKY22/29 and WRKY25/33, accompanied by biosynthesis of stress-related hormones such as ethylene and jasmonic acid (Chinchilla *et al.*, 2007; Boller and Felix, 2009; Chang and Nick, 2012; Guan *et al.*, 2015). Salicylic acid, a central player in the hypersensitive response (ETI) to biotrophic pathogens, seems to be dispensable for basal immunity: in the grapevine cell system used in the current study, SA was found to yield only 10% of the phytoalexin accumulation seen for induction by the jasmonate pathway (Chang *et al.*, 2017). These elicitor-triggered defence responses can be manipulated by effectors secreted by the pathogen. Pathogen effectors can support infection success by indirectly modulating targets outside of innate immunity (Pelgrom and Van den Ackerveken, 2001).

Thus, the outcome of a pathogen–host encounter depends on chemical signals (any molecules or events that confer information) that are secreted by the invader and these are subject to evolutionary change. Formally, there exist three modes of action of such chemical signals: (i) elicitor (such as a PAMP) activation of host defence, (ii) effector silencing of host defence after it had been activated by an elicitor, and (iii) effector negative regulation of elicitor-triggered defence. It is conceivable that positive regulators exist that by themselves cannot elicit anything, but promote host defence once it has been activated by elicitors. This possibility seems to be counter-intuitive at first glance. What selective advantage should a pathogen draw from even further amplifying host defence? However, in the natural context, a pathogen is seldom alone, but has to compete with other pathogens for the host’s resources. Since attacked host cells will activate chemical warfare to ward off the intruders, a pathogen can acquire mechanisms to evade or degrade such host compounds and, thus, by stimulating the defence response of the host, outcompete its rival without the need for direct attack, representing a kind of plant version of a famous Chinese war trick (借刀杀人 *jiè dāo shā rén*, ‘kill somebody with a borrowed sword’; Sun, 1991). We will in the following use the term *amplifier* for this type of immunity-modulating signal.

Such pathogen consortia can have enormous economic impact, as seen for the grapevine trunk diseases (GTDs), which are a major problem in viticulture and the wine industry because they diminish vineyard longevity and productivity and cause tremendous economic losses worldwide (Bertsch *et al.*, 2013; Gramaje *et al.*, 2018; Mondello *et al.*, 2018). To date, as many as 133 fungal species, belonging to 34 genera, have been reported to be associated with the GTD syndrome (Gramaje *et al.*, 2018). Surprisingly, the same fungi can also be isolated from healthy grapevine trunks and in similar frequencies to those of diseased grapevines but without causing disease symptoms; as such they are viewed as latent pathogens (Verhoeff, 1974; González and Tello, 2011; Hofstetter *et al.*, 2012). In addition to these fungi, bacteria inhabit the woody tissues of both asymptomatic and GTD-diseased grapevine, and might interact with the fungal colonizers in a complex and unknown manner (Bruez *et al.*, 2015). Whether this microbial endoflora will lead to disease or remain latent depends on the stress status of the host. Especially heat and drought stress, but also the host genotype, are relevant in this context (Graniti *et al.*, 2000; Surico *et al.*, 2006). The devastating damage caused by this type of disease is also due to the symptoms progressing slowly, often over many years, and then breaking out suddenly, leading to a so-called apoplectic breakdown, in which the grape dies within a few days, often at the time when its productivity is maximal (Gramaje *et al.*, 2018).

One of the central fungi seems to be *Eutypa lata*, causing one of the most the severe GTDs, Eutypa dieback (Mutawila *et al.*, 2017). The fungus generally infects the vine through pruning wounds, and causes brown sectorial necrosis leading to stunted vegetative growth (Moisy *et al.*, 2017). GTDs obviously differ from the classical case of infectious diseases following Koch’s postulates (actually first published by his disciple: Loeffler, 1884). The disease is not caused by the mere presence of a particular microbial organism, but by a change of its behaviour. In other words, there must be signals that are exchanged between pathogen and host that determine the outbreak of GTD symptoms.

To be able to monitor the effect of immunity ‘modulators’ (effectors or amplifiers) in cell culture would require that, first, basal immunity (PTI) is activated. One of the first cellular defence responses is a remodelling of the cytoskeleton (for a recent review, see Park *et al.*, 2018). Also, in grapevine, rapid elimination of microtubules (Qiao *et al.*, 2010) and rapid contraction of actin filaments (Guan *et al.*, 2014) are among the earliest defence responses. The fact that several pathogen effectors specifically target microtubule or microtubule-associated proteins supports a central role of the cytoskeleton in defence (Lee *et al.*, 2012; Guo *et al.*, 2016).

In the current study, we searched for fungal amplifiers by screening the effect on grapevine defence of culture filtrates from fungi involved in GTDs, based on the assumption that these extracts have abundant general elicitors, and that a strong induction of defence will indicate the presence of amplifiers. By comparing two extracts originating from the same strain of *E. lata* that contrast with respect to their defence-inducing activity, we pursued a bioactivity-guided fractionation strategy leading to the identification of *O*-methylmellein. Using the bacterial PAMP flg22 as a platform to trigger PTI, we show that *O*-methylmellein qualifies as an amplifier in the context of flg22-triggered PTI.

## Materials and methods

### Cell culture and chemicals

Suspension cell cultures of *V. rupestris* expressing the fluorescent tag green fluorescent protein (GFP) linked with the tubulin marker *AtTUB6* (Guan *et al.*, 2015), and *V. vinifera* L. cv. ‘Chardonnay’ expressing the actin marker *Fimbrin Actin-Binding Domain 2* (*FABD2)–GFP* were used in this experiment and cultivated as described previously (Wang and Nick, 2017; Akaberi *et al.*, 2018). If not stated otherwise, all these treatments were conducted with cells at the onset of the expansion phase (day 4 after subcultivation).

The peptide flg22 was synthesized by a commercial provider (GenScript) and diluted in sterile H_2_O. Diphenylene-iodonium chloride (DPI; Sigma-Aldrich, Deisenhofen, Germany), an inhibitor of NADPH oxidase respiratory burst oxidase homologue (RboH) (Bolwell and Wojtaszek, 1997), was prepared in dimethylsulfoxide (DMSO). GdCl_3_, an inhibitor of calcium influx (Ding and Pickard, 1993), was prepared in distilled water. The commercially available elicitor harpin (Messenger; EDEN Bioscience Corp., Bothell, WA, USA), which induces significant cell death in *V. rupestris* cells, was diluted in distilled water (Chang and Nick, 2012).

### Culture extracts from grapevine trunk disease-related fungi

Culture filtrate extracts of *Phaeomoniella chlamydospora* (yeast malt glucose agar; HMG), *Phaeoacremonium minimum* (HMG), *Eutypa lata* (HMG), *Fomitiporia mediterranea* (HMG), *Botrytis cinerea* (HMG), *Roesleria subterranea* (HMG), *Guignardia bidwellii* (HMG), *Eutypa lata* Institut für Biotechnologie und Wirkstoff-Forschung (IBWF) E16012 (biotin–aneurin–folic acid agar; BAF), *Eutypa lata* IBWF E16012 (potato dextrose agar; PDA), *Eutypa lata* 5.1 (BAF), *Eutypa lata* 5.1 (PDA), *Eutypa lata* 5 (BAF), *Eutypa lata* 5 (PDA), *Eutypa lata* Hanns-Heinz Kassemeyer (HKM) 2 (BAF), *Eutypa lata* HKM2 (PDA), all of which are associated with trunk diseases of grapevine, were prepared as follows.

#### Fungal strains and culture conditions

*Phaeomoniella chlamydospora* CBS 229.95 (*Pch*) and *Phaeoacremonium minimum* (*Togninia minima*) CBS 100398 (*Pmi*) were purchased from the CBS-KNAW culture collection (CBS-KNAW Fungal Biodiversity Centre, The Netherlands) (Markwart *et al.*, 2019). The *Botrytis cinerea* strain was maintained as previously published (Schüffler *et al.*, 2009). *Guignardia bidwellii* was obtained from the Centraalbureau voor Schimmelcultures (CBS, Fungal Biodiversity Centre, The Netherlands) as described previously (Buckel *et al.*, 2017). The *Eutypa lata* strains (except for the IBWF E16012) were kindly provided by apl. Prof. Dr Hanns-Heinz Kassemeyer (Staatliches Weinbauinstitut Versuchs und Forschungsanstalt für Weinbau und Weinbehandlung). The *Eutypa lata* strain IBWF E16012 is part of the IBWF culture collection and was isolated by Linda Muskat in a Hessian vineyard. The *Roesleria subterranea* strain 1303-K is also part of the IBWF culture collection and was provided by Isabell Büttel. For maintenance the fungal strains were cultured on HMG agar (4 g l^−1^ yeast extract, 10 g l^−1^ malt extract, 10 g l^−1^ glucose, 2% agar, pH 6.5) or BAF agar (0.1% yeast extract, 1% glucose, 2% maltose, 0.2% peptone, 0.05% KH_2_PO_4_, 0.04% MgSO_4_^.^7H_2_O, 0.007% CaCl_2_^.^2H_2_O, 0.001% FeCl_3_^.^6H_2_O, 0.0002% ZnSO_4_) or PDA (dehydrated mashed potatoes 2% w:v, glucose 2% w:v, pH 5.5), and were transferred to new agar plates every 2–4 weeks. The fungi were cultivated in 500 ml HMG in 1-litre Erlenmeyer flasks on an orbital shaker (120 rpm, 22±1 °C) (Kramer *et al.*, 2009). The availability of free glucose within the medium was monitored by Diabur test 5000 strips (Roche, Germany). Once the free glucose in the medium was depleted, the fermentation was stopped by separating culture broth from the mycelium by filtration.

#### *Isolation*, *HPLC and HPLC-MS analysis*, *internal standards*, *and compound library*

Culture filtrates were extracted with 1 vol. ethyl acetate, and concentrated 1:100 *in vacuo*. Aliquots of 400 µg of the extracts were fractionated by HPLC (Series 1100, Hewlett–Packard, Waldbronn, Germany; equipped with a LiChrospher RP18 column; 5 µm, 125 mm×4 mm, Merck, Darmstadt, Germany) with a 0.1% v:v formic acid: MeCN gradient (1% to 100% MeCN in 20 min; flow: 1 ml min^−1^) for bioactivity-guided fractionation as described previously (Buckel *et al.*, 2013). The fractionated extracts and pure compounds obtained from these small-scale separation processes were re-analysed for their biological activity. Subsequently, the fractions from five runs were pooled (corresponding to 2 mg of extract) for those extracts that had been prioritized by their bioactivity into 96-well plates and dried *in vacuo*. To identify the bioactive compounds, a HPLC-MS (Series 1200, Agilent, Waldbronn, Germany) equipped with an UV-DAD, and a coupled LC/MSD trap atmospheric pressure chemical ionization mass spectrometer with positive and negative polarization were used. Methods were applied as described previously (Buckel *et al.*, 2017). The MS spectrum information for molecules and HPLC-MS analysis results are respectively given in Supplementary Fig. S1 and Supplementary Table S1.

#### Isolation of O-methylmellein

After the fermentation of *Eutypa lata* IBWF E16121 in 20 litres BAF medium, 12 litres culture filtrate was obtained and extracted with 1 vol. diethyl ether. One gram of isolated extract was separated by Chromabond® C18ec solid phase extraction with 20% MeCN in H_2_O. The intermediate was further purified by preparative HPLC (Jasco PU-2087 Plus, Jasco Labor- und Datentechnik, Gross-Umstadt) using the following method: 65% MeCN in H_2_O for 10.7 min; flow: 13.65 ml min^−1^; column: SunFire™ C18 5 µm, 250 mm×19 mm (Waters GmbH, Eschborn). From this preparative run, 65.5 mg *O*-methylmellein were obtained. The structure was clarified by the working group of Prof. Dr Helge B. Bode, Goethe University Frankfurt.

### Extracellular alkalinization

Alkalinization of the culture medium by *V. rupestris* cells expressing the fluorescent tubulin marker GFP–*At*TUB6 was measured as described in Qiao *et al.* (2010). The pH changes were recorded over time, and values for ΔpH were calculated as differentials of treatment versus mock control using the peak values to estimate ΔpH_max_. The experiments were repeated at least five times.

### RNA extraction and cDNA synthesis

Following the various treatments, total RNA was purified using the Universal RNA Purification Kit (Roboklon, Berlin, Germany), according to the protocol of the producer. The cDNA synthesis followed the protocol in Wang *et al*. (2019).

### Semi-quantitative PCR

Semi-quantitative PCR (semi-qPCR) was performed as described previously (Duan *et al.*, 2015). The PCR was performed using *Taq* polymerase from New England Biolabs (Frankfurt, Germany). Each experiment was repeated with three biological replicates, each in three technical replicates.

Semi-qPCR results were quantified by quantitative image analysis using the freeware ImageJ (https://imagej.nih.gov/ij/) from the digital images recorded for the electrophoretically separated amplicons. For each band, the integrated density was measured along a probing line transecting the band, and the results integrated into an Microsoft Office Excel spreadsheet. The methanol solvent control was used as the internal standard for relative quantification of the other bands on the gel. For visualization of the complex and extensive datasets, relative changes of induction levels were calculated as the relative surplus of expression for the fraction from the BAF supernatant over the corresponding fraction from the PDA supernatant. These were encoded in a colour code from dark blue (inhibited response) to dark red (enhanced response) and plotted along with the elution profile (Fig. 3).

### Real-time PCR analysis

Quantitative real-time PCR (qRT-PCR) was conducted using a CFX96^TM^ real-time PCR cycler (Bio-RAD, USA) as described previously (Wang *et al*., 2019). Data analysis was performed using the 2^–∆∆*C*^_t_ method (Livak and Schmittgen, 2001). The ‘fold control’ in all gene expression figures signifies the comparison between transcript levels of genes in the chemical treatment and respective control treatment (considered as ‘1’). Significance analysis was tested by using Student’s *t*-test in Microsoft Office Excel at the 0.05, 0.01, and 0.001 levels. Each experiment was repeated with three biological replicates, each in three technical replicates.

The elongation factor 1α was used as an internal standard to quantify the transcript levels of different genes. The primers used in semi-qPCR and qPCR are given in Supplementary Table S2.

### Life-cell visualization of the cytoskeleton

Making use of the GFP tag linked to either the tubulin marker *At*TuB6, or the actin marker FABD2, the cytoskeleton could be monitored in living cells of grapevine. The responses of cortical microtubules and actin filaments were monitored over time in individual cells by spinning-disc confocal microscopy. Confocal z-stacks were recorded as described in Wang and Nick (2017).

To quantify the integrity of actin organization, a strategy modified from Schwarzerová *et al.* (2002) was used, based on intensity profiles collected by ImageJ software (https://imagej.nih.gov/ij/) along a probing line of 10 pixel width and spline averaging to correct for random fluctuations over subsequent frames from a time-lapse series. Since depletion of the network will reduce the amplitude of the peaks and widen the intensity of troughs between the peaks, the standard error over the profile can be used as the readout for integrity/intensity of the network. We used the relative change ΔInt compared with the first frame of the series as the readout for microtubular disintegration or actin depletion, respectively. Quantitative analysis of the relative responses of cortical microtubules and actin filaments represented at least three independent experimental series with a population of at least 50 individual cells. Significance analysis was tested by Student’s *t*-test in Microsoft Office Excel at the 0.05, 0.01, and 0.001 levels.

### Determination of mortality

To determine mortality, the Evans Blue (Sigma-Aldrich) dye exclusion test was used as described in Chang and Nick (2012). At least 1000 cells were scored per sample. The ratio of the dead cells over the scored cells was calculated as the cell death frequency. Significance analysis was tested by using Student’s *t*-test in Microsoft Office Excel at the 0.05, 0.01, and 0.001 levels. Data represent a population of 3000 cells scored over three independent experiments.

## Results

### The response to fungal culture filtrates is associated with the expression of basal immunity

To detect amplifiers, we screened for activation of defence responses based on the assumption that accompanying elicitors should be present in the extracts. As a rapid readout for defence, we monitored changes of extracellular pH, since activation of the defence-related calcium influx through proton co-import will lead to apoplastic alkalinization (Jabs *et al.*, 1997; Felix *et al.*, 1999; Nürnberger and Scheel, 2001). This pH shift has been shown for our experimental system to be blocked by Gd^3+^, an inhibitor of calcium channels (Qiao *et al.*, 2010), corroborating that it can be used as a readout. To find candidate ‘pools’ of defence modulators, we tested the effects of fungal filtrates on the *V. rupestris* GFP–*At*TUB6 cell line by measuring alkalinization of the culture medium. The cells were incubated with 15 culture extracts (the respective media are given in parentheses in Materials and methods, ‘Culture extracts from grapevine trunk disease-related fungi’) from fungus-associated grapevine diseases, with a focus on GTD, particularly *E. lata* (Supplementary Table S3). In most treatments, the pH increased rapidly after the addition of these extracts. The response to *E. lata* ranged from less than 0.05 to more than 0.5 units, depending on the tested strain, indicative of considerable intraspecific variability (Supplementary Table S3).

**Fig. 1.**
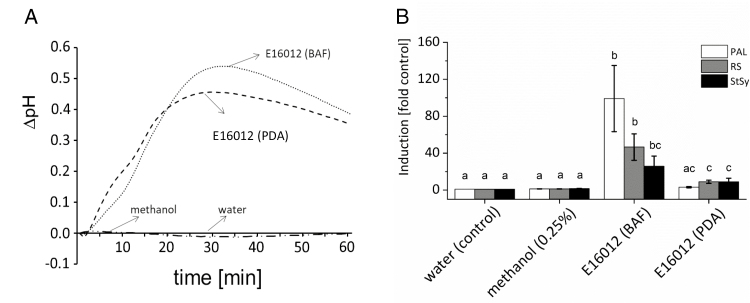
*Eutypa lata* IBWF E16012 culture extracts activate extracellular alkalinization and phytoalexin-synthesis gene induction in the cells of *V. rupestris* expressing the fluorescent tubulin marker GFP–*At*TUB6. (A) The extracellular ∆pH of the tubulin marker cell line (*V. rupestris* TuB6) to 25 µg ml^−1^ filtrates from E16012 BAF agar (BAF) and potato dextrose agar (PDA) was recorded by pH meter, with 0.25% methanol as the solvent control and water as the control. (B) The expression of phytoalexin-synthesis genes (*PAL*, phenylalanine ammonia lyase; *RS*, resveratrol synthase; *StSy*, stilbene synthase) to the same treatments for 1 h was measured by qPCR using elongation factor 1α as an internal standard. The fold induction of defence genes in each treatment as compared with water control (considered as ‘1’) was analysed using Microsoft Office Excel. Error bars indicate standard error of the mean. Different lowercase letters show significance at *P*=5%, n=3.

As a second readout for defence, the activation of phytoalexin-synthesis genes was monitored. All filtrates activated a strong increase in steady-state transcript levels for *PAL* (phenylalanine ammonia lyase), *RS* (resveratrol synthase), and *StSy* (stilbene synthase) (Supplementary Fig. S2). Among those samples, a particular pair was interesting: for *E. lata* IBWF E16012, these transcripts (*PAL*, *RS*, and *StSy*) were strongly induced during cultivation with BAF medium, while the same strain produced a much lower transcript level (Supplementary Fig. S2, black and white arrows) when cultivated on PDA. Interestingly, the extracellular alkalinization induced by these two extracts was similar (Fig. 1A). Nevertheless, the response of phytoalexin genes differed, whereby the response to culture filtrate obtained from BAF-cultivated fungi was much stronger than that seen for PDA. Although the transcript induction in PDA was small, there still were some differences; for instance, compared with the water control, the relative transcription of genes *PAL*, *RS*, and *StSy* in PDA treatment was 3.2, 9.0, and 8.9, respectively (Fig. 1B). The response to culture filtrate obtained from BAF-cultivated fungi was much stronger than that seen for PDA and the amplitude of this difference was dependent on the investigated transcript. We therefore decided to use this contrasting pair of culture filtrates for activity-guided fractionation of immunity modulators, because here the same fungal strain obviously produced quite different immunity modulator activities, depending on the conditions.

**Fig. 2.**
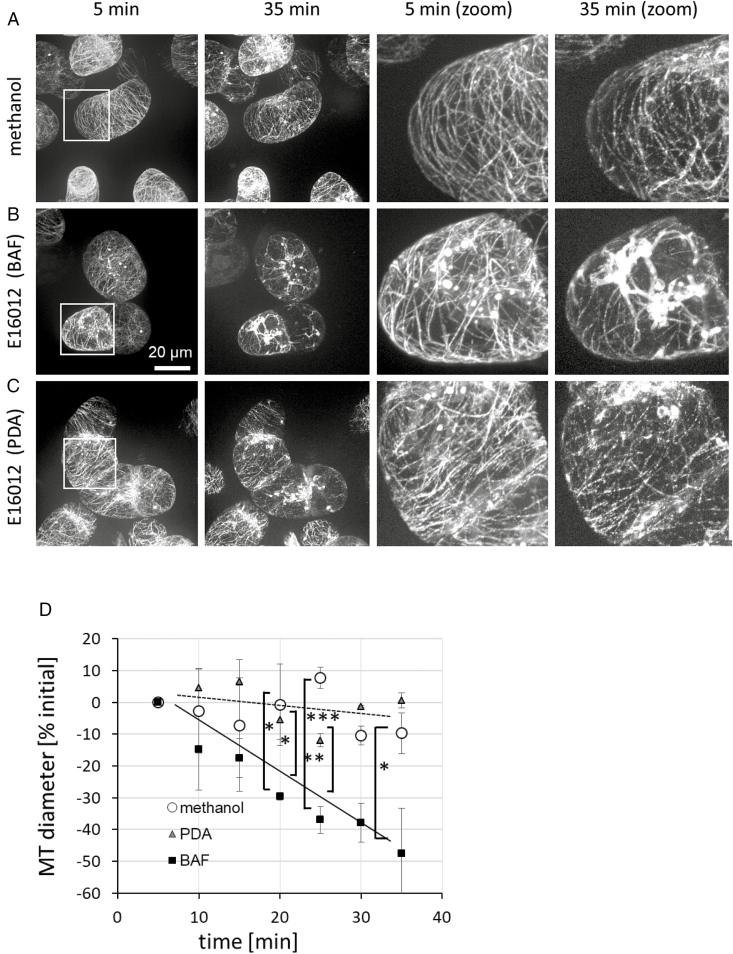
Microtubular responses to culture filtrates of *Eutypa lata* IBWF E16012 in the *V. rupestris* microtubule marker line GFP–*At*TuB6. Microtubules in the cells of *V. rupestris* with 0.25% methanol solvent control (A), 25 µg ml^−1^ E16012 (BAF) filtrate (B), and 25 µg ml^−1^ E16012 (PDA) filtrate (C) for 5 and 35 min were observed by spinning disc microscopy. An enlargement of the microtubules is also shown. (D) The alteration of microtubule (MT) thickness in relative units over the time course (5, 10, 15, 20, 25, 30, 35 min) of the treatments. Error bars signify standard error of the mean; *P<0.05, **P<0.01, ***P<0.001 (Student’s *t*-test), *n*=3. Observations were representative of at least four independent experimental series with a population of 50 individual cells for each treatment. Scale bar: 20 μm.

The reorganization of host cell microtubules has been reported as a rapid response to infection by oomycetes or fungi (Hardham *et al.*, 2008; Hoefle *et al.*, 2011). To test whether secondary metabolites secreted by *E. lata* IBWF E16012 can evoke a microtubular response, we followed microtubules after the addition of the two culture filtrates. For the treatments of PDA extract and solvent control, microtubules became thinner and less continuous, but maintained their overall integrity (Fig. 2A, C). In contrast, microtubules had significantly disintegrated and strong aggregations of fluorescence signals appeared in the cell centre in response to BAF extract (Fig. 2B). Quantification of the microtubule response over time (Fig. 2D) showed around 10% reduction in average microtubule diameter (caused by the gaps of microtubule continuity) for the treatment with solvent or PDA extract, while for BAF extract, the score dropped to around half of the initial value. Thus, the extract from BAF-cultivated *E. lata* was active not only with respect to phytoalexin-related transcripts, but also with respect to microtubule remodelling.

### Activity-guided fractionation of *E. lata* IBWF E16012 filtrates leads to the isolation of *O*-methylmellein

Since the strain *E. lata* IBWF E16012, if cultivated in BAF medium, secreted compounds that efficiently activate phytoalexin transcripts along with a microtubular response, while the same strain did not produce this bioactivity when cultivated in PDA medium, we used these contrasting conditions of the same biological material for a bioactivity-guided fractionation strategy.

**Fig. 3.**
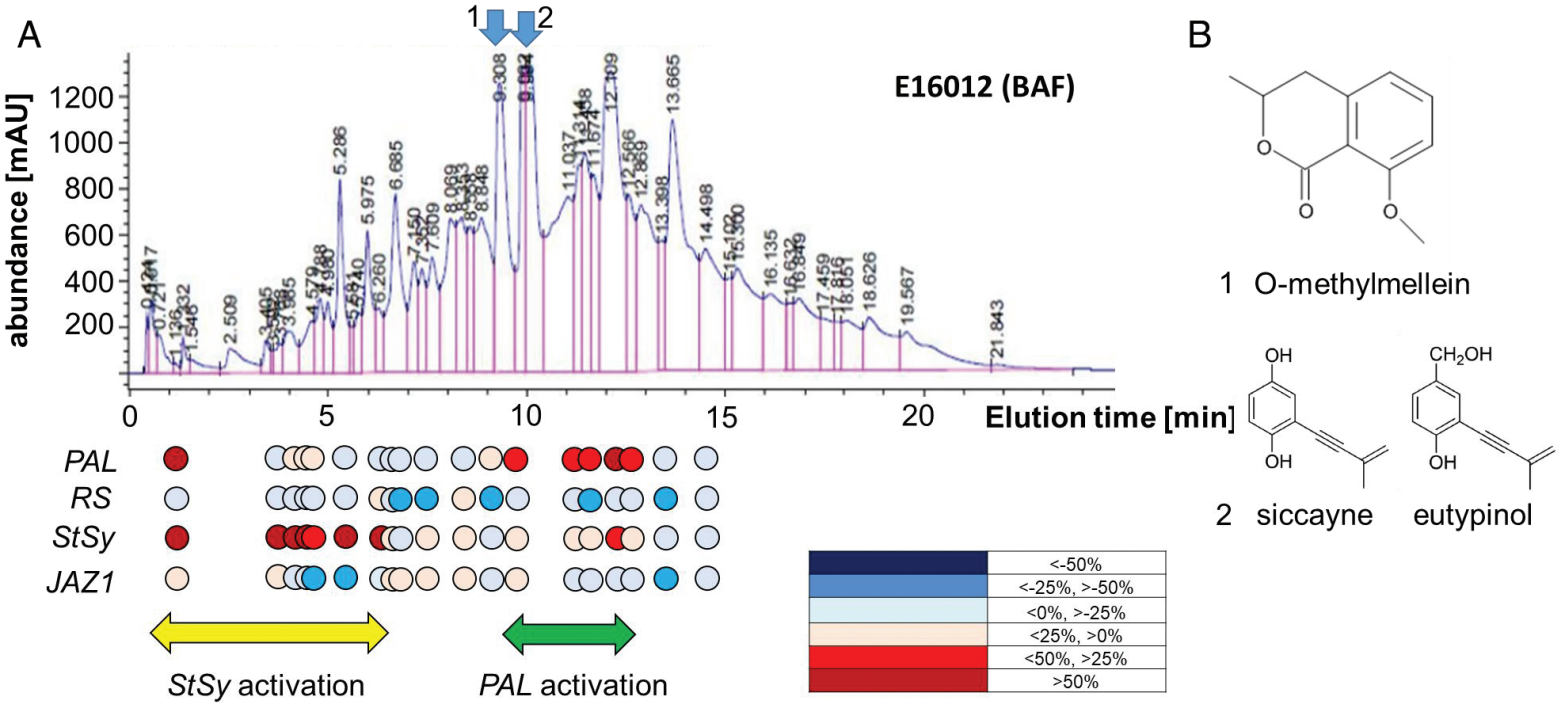
Fractionation of the *Eutypa lata* IBWF E16012 (BAF) culture filtrate and the effect of fractions on the expression of phytoalexin genes in the cell line *V. rupestris* GFP–*At*TuB6. (A) E16012 (BAF) culture filtrate fractionated by HPLC. The coloured circles show Δ (BAF–PDA): PDA as a percentage showing the stimulation due to medium change and elution time interpolated from the two runs. The cells were treated for 1 h with 20 μl solution of HPLC fractions obtained from the *E. lata* IBWF E16012 (BAF) and the *E. lata* IBWF E16012 (PDA) extracts or 0.2% methanol as a mock control; the transcription of genes *PAL*, *RS*, *StSy*, and *JAZ1* was measured by semi-qPCR and the semi-qPCR results were quantified by ImageJ. (B) Secondary metabolites identified from *E. lata* IBWF E16012 extracts: (1) *O*-methylmelleim; (2) siccayne and eutypinol. The relative induction levels were expressed by using different colours: dark blue: <−50%; blue: <−25%, >−50%; light blue: <0%, >−25%; light red: <25%, >0; red: <50%, >25%; dark red: >50%.

We therefore fractionated the two filtrates IBWF E16012 (BAF) and IBWF E16012 (PDA) by preparative HPLC, and collected the fractions in 96-well plates. The bioactivity of these fractions was then assessed by measuring steady-state transcript levels of genes involved in phytoalexin synthesis (*PAL*, *RS*, *StSy*) along with *JAZ1* as a readout for the activity of basal immunity (Chang *et al.*, 2017) in *V. rupestris* cells as a reporter. To identify those fractions that differed in bioactivity between the two culture filtrates, the gene expression obtained for the fraction generated from the more active BAF-derived extract was calibrated against the value observed for the same fraction in the less active PDA-derived extract. When these changes in bioactivity were projected upon the elution profiles (Fig. 3A), two major hotspots of bioactivity emerged: (i) the fractions eluting earlier than 6.5 min produced stronger activation of *StSy*, if these fractions originated from the BAF culture filtrate; and (ii) the fractions eluting between 9 and 13 min produced a stronger activation for *PAL* (to a lesser extent also of *StSy*), if coming from the BAF culture filtrate. Since *PAL* was the gene activity for which the bioactivity of the two cultures contrasted most (Fig. 1B), the second hotspot was then subjected to closer scrutiny, searching for molecular components that might correlate with bioactivity.

In several cases, the identification of molecular candidates correlating with gene activation was achieved by subsequent mass spectrometry (Fig. 3). Of special interest was a peak eluting at 9.05 min, because this peak was significantly larger in the BAF elution profile (1250 mAU) than in the PDA profile (950 mAU). The lead compound identified in this peak was *O*-methylmellein (Fig. 3A, B, 1; Supplementary Fig. S3). The subsequent peak, eluting between 9.6 and 9.8 min, was instead more similar between the two culture filtrates and contained siccayne and eutypinol (Fig. 3A, B, 2; Supplementary Fig. S3). Additional molecular candidates such as FS E16012-4, FS E16123-1, FS E16123-3, and FS E16123-5 were isolated, but could not be structurally elucidated (Supplementary Table S3).

Since the peak harbouring *O*-methylmellein differed significantly in abundance between the corresponding fractions from the two culture filtrates, and since this difference was correlated with a differential activation of *PAL*, as first committed step of the phenylpropanoid pathway giving rise to lignin as well as to stilbenes, we wondered whether *O*-methylmellein acted as an elicitor, or whether it might act, in concert with other compounds present in the fraction, as an amplifier.

### *O*-Methylmellein is not acting as an elicitor

**Fig. 4.**
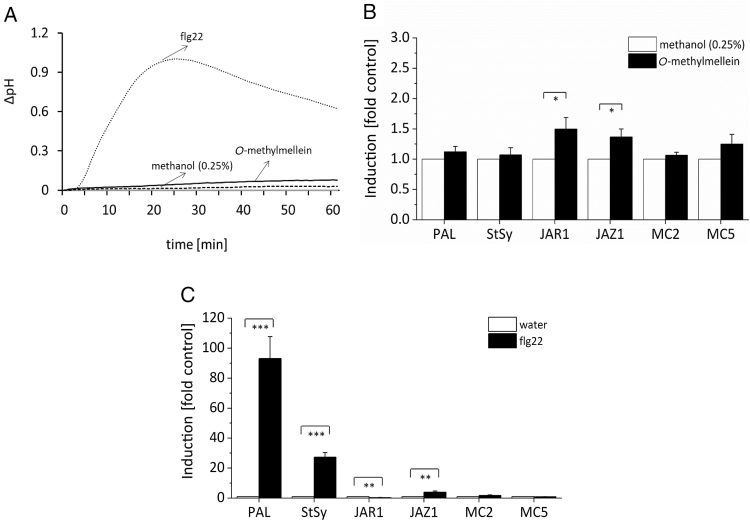
Effect of *O*-methylmellein and flg22 on extracellular alkalinization and induction of defence genes in the *V. rupestris* microtubule marker line GFP–*At*TuB6. (A) Cells were challenged with 25 µM *O*-methylmellein, 1 µM flg22 as the positive control, or 0.25% methanol as the solvent control. Extracellular ∆pH was recorded by pH meter. (B) Cells were elicited by 25 µM *O*-methylmellein for 1 h, with 0.25% methanol as solvent control. (C) Cells were treated with 1 µM flg22 for 1 h, with distilled water used as control. The steady-state transcript levels were measured by real time qPCR for *PAL* (as readout for the activation of the phenylpropanoid pathway), *StSy* (as readout for the activation of the phytoalexin-branch of this pathway), *JAZ1* (as readout for the jasmonate signalling monitoring basal immunity), *JAR1* (as readout for synthesis of active jasmonate), and the metacaspases *MC2* and *MC5* (as readouts for cell-death-related defence). Values for relative transcript abundance were calculated using elongation factor 1α as an internal standard. The induction levels of defence genes in *O*-methylmellein and flg22 treatments were compared with 0.25% methanol (B) (considered as ‘1’), or water control (C) (considered as ‘1’), respectively. The values were analysed with Microsoft Office Excel. Error bars indicate standard error of the mean; *P<0.05, **P<0.01, ***P<0.001 (Student’s *t*-test), *n*=3.

If *O*-methylmellein were active as an elicitor, it should, when administered as a pure compound, induce hallmarks of basal immunity, such as activation of calcium influx or activation of phytoalexin synthesis genes as compared with other defence-related genes. Firstly, we measured the effect of different concentrations of *O*-methylmellein and corresponding concentrations of the solvent methanol on extracellular alkalinization in *V. rupestris* cells (Supplementary Fig. S3). *O*-Methylmellein could not induce any extracellular alkalinization (Supplementary Fig. S3), which is in stark contrast to the strong pH response seen for the original total extract (Fig. 1). In order to validate this finding, the bacterial elicitor flg22, a well-studied PAMP whose signalling pathway has been dissected in great detail, was used as a positive control to trigger extracellular pH change (Chang and Nick, 2012). As expected, flg22 triggered a rapid and significant extracellular alkalinization, up to 1.0 in 25 min. However, *O*-methylmellein only induced a much weaker pH change, around 0.05 (Fig. 4A).

To test, whether *O*-methylmellein would induce defence-related genes, we measured steady-state transcript levels by real-time qPCR for *PAL*, *StSy*, *JAZ1*, *JAR1*, and the metacaspases *MC2* and *MC5* in response to *O*-methylmellein. There was no significant induction for any of these genes, except a slight elevation for *JAZ1* and *JAR1* indicative of a mild activation of basal immunity (Fig. 4B). This was in sharp contrast with the strong activation of *PAL* seen for the supernatant from the BAF culture (Fig. 1B), and the still considerable induction of this gene observed by the respective HPLC fraction (induction factor over solvent control 4.6±0.534, *n*=3). Flg22 also was used as a positive control for an elicitor of PTI in comparison with *O*-methylmellein. This positive control induced significant induction of defence genes, such as *PAL*, *RS*, *StSy*, and *JAZ1*, but not *MC2* and *MC5* (Fig. 4C). Thus, *O*-methylmellein can induce neither calcium influx nor any significant response of defence-related genes, and therefore does not qualify as an elicitor.

### *O*-Methylmellein amplifies flg22-triggered induction of phytoalexin-synthesis genes, but not extracellular alkalinization

**Fig. 5.**
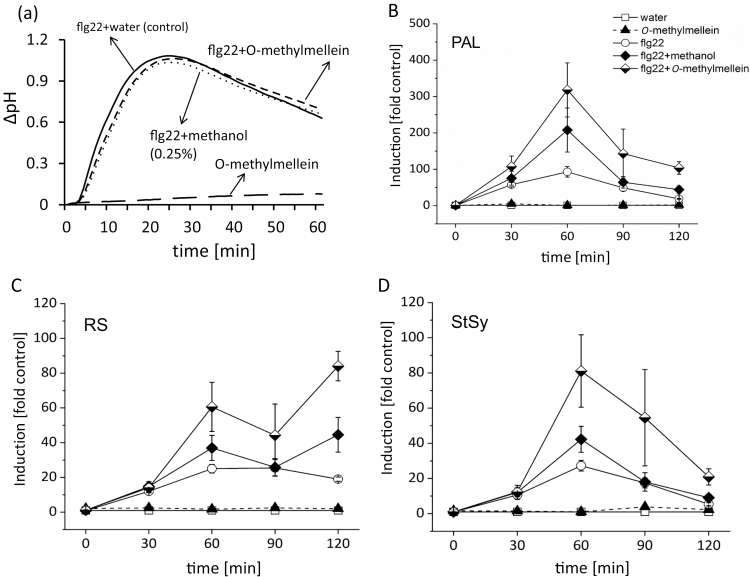
Modulatory effect of *O*-methylmellein on the flg22-triggered extracellular alkalinization and defence gene transcription in the cell line *V. rupestris* GFP–*At*TuB6. (A) The extracellular pH responses to 25 µM *O*-methylmellein alone, or a combination of 1 µM flg22 with water control, 0.25% methanol solvent control, or 25 µM *O*-methylmellein was recorded over time. (B–D) The induction of phytoalexin-synthesis genes under treatment with water, 25 µM *O*-methylmellein, 1 µM flg22, 1 µM flg22 combined with 0.25% methanol or 25 µM *O*-methylmellein for indicated time points (0, 30, 60, or 120 min). The transcription level of genes *PAL* (B), *RS* (C), and *StSy* (D) was measured by qPCR using elongation factor 1α as an internal standard and compared with water control (considered as ‘1’) at each time point. The data were analysed with Microsoft Office Excel. Error bars indicate standard error of the mean, *n*=3.

Some compounds secreted by the pathogen might affect plant immune response indirectly, rather than activating the first level of plant immunity. To test, whether *O*-methylmellein is able to regulate the basal immune responses stimulated by flg22, we used the same strategy as described above and examined extracellular alkalinization along with transcript levels of phytoalexin synthesis genes (Fig. 5), but administered *O*-methylmellein in combination with flg22. As was to be expected, flg22 induced a significant ΔpH response, up to 1.06 in 25 min (Fig. 5A), but there was no difference when it was combined with *O*-methylmellein or methanol control. Therefore, *O*-methylmellein does not modulate flg22-triggered extracellular alkalinization in cells of *V. rupestris*.

Nevertheless, *O*-methylmellein might, by activation of a pathway that is independent of calcium, modulate the expression of phytoalexin-synthesis genes. Using the same experimental design, the expression of *PAL*, *RS*, and *StSy* was followed over time. We found that flg22 could trigger the transcripts for all three genes. In combination with methanol, this induction was slightly, but significantly, enhanced (Fig. 5). Interestingly, *O*-methylmellein by itself failed to cause any induction of defence genes (Fig. 5) but strongly enhanced the response of this transcript to flg22: *O*-methylmellein in combination with flg22 induced *PAL* transcripts 318-fold, much stronger than the 208-fold induction seen for methanol with flg22 or the 93-fold induction obtained for flg22 alone (Fig. 5B). Similar enhancements were observed for the other genes, albeit with a different temporal pattern for *RS* (Fig. 5C). For *StSy*, the combined treatment of *O*-methylmellein with flg22 reached an induction of 81-fold at 60 min, as compared with 42-fold for flg22 with methanol and 27-fold for flg22 alone (Fig. 5D). Irrespective of the tested gene and the amplitude of the response to flg22 alone, *O*-methylmellein enhanced the amplitude of the response to flg22 by a factor of 3. Thus, *O*-methylmellein interacts with flg22 in a multiplicative manner. This interaction occurs on the functional not the physical level.

### The amplifier activity of *O*-methylmellein is independent of calcium influx, but dependent on RboH

**Fig. 6.**
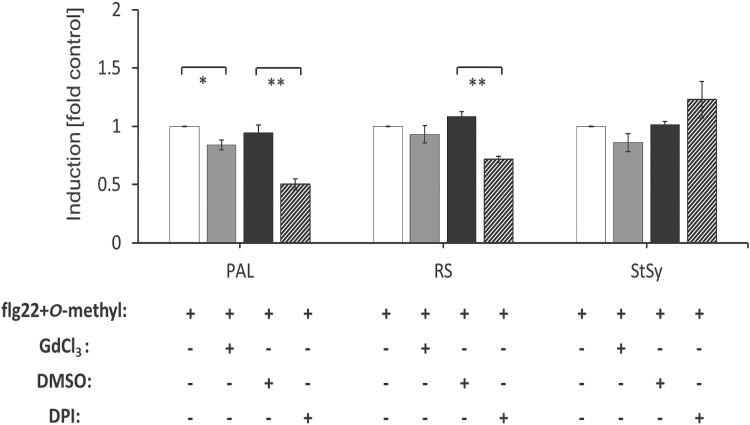
Roles of Gd-sensitive calcium channels and reactive oxygen species in the *O*-methylmellein (*O*-methyl)-modulated defence gene transcription triggered by flg22 in the cell line *V. rupestris* GFP–*At*TuB6. Cells were challenged by the combination of 1 µM flg22 and 25 µM *O*-methylmellein alone, or with 100 µM GdCl_3_ (the calcium channel blocker), 10 µM DPI (the NADPH oxidase inhibitor), or 0.1% DMSO as solvent control of DPI for 1 h. The induction of the genes *PAL*, *RS*, and *StSy* was measured by qPCR. Values for relative transcript abundance were analysed using elongation factor 1α as an internal standard and compared with flg22 and *O*-methylmellein treatment (considered as ‘1’) with Microsoft Office Excel. Error bars indicate standard error of the mean; *P<0.05, **P<0.01 (Student’s *t*-test), *n*=3.

To further explore the amplification of flg22 triggered defence signalling by *O*-methylmellein, we either blocked calcium influx by GdCl_3_ or inhibited the NADPH oxidase RboH by DPI prior to the co-treatment of *O*-methylmellein and flg22. *O*-Methylmellein by itself did not induce any of the tested genes (Fig. 5, triangles, dashed lines), while flg22 by itself produced an induction between 20- and 100-fold, depending on the gene (Fig. 5, open circles, solid lines). When the calcium channels were blocked by GdCl_3_, the induction of the three genes was only slightly suppressed (Fig. 6). In contrast, the transcription of *PAL* and *RS* was strongly inhibited by DPI, but this inhibition was not observed in the case of *StSy* (Fig. 6). Thus, on the level of phytoalexin-gene expression, the amplification of the flg22 response by *O*-methylmellein is mostly independent of calcium influx, for all three genes. Instead, the expression of *PAL* and *RS* is strongly dependent on RboH activity (which is not the case for *StSy*).

### *O*-Methylmellein amplifies flg22-triggered disassembly of actin, but not of microtubules

**Fig. 7.**
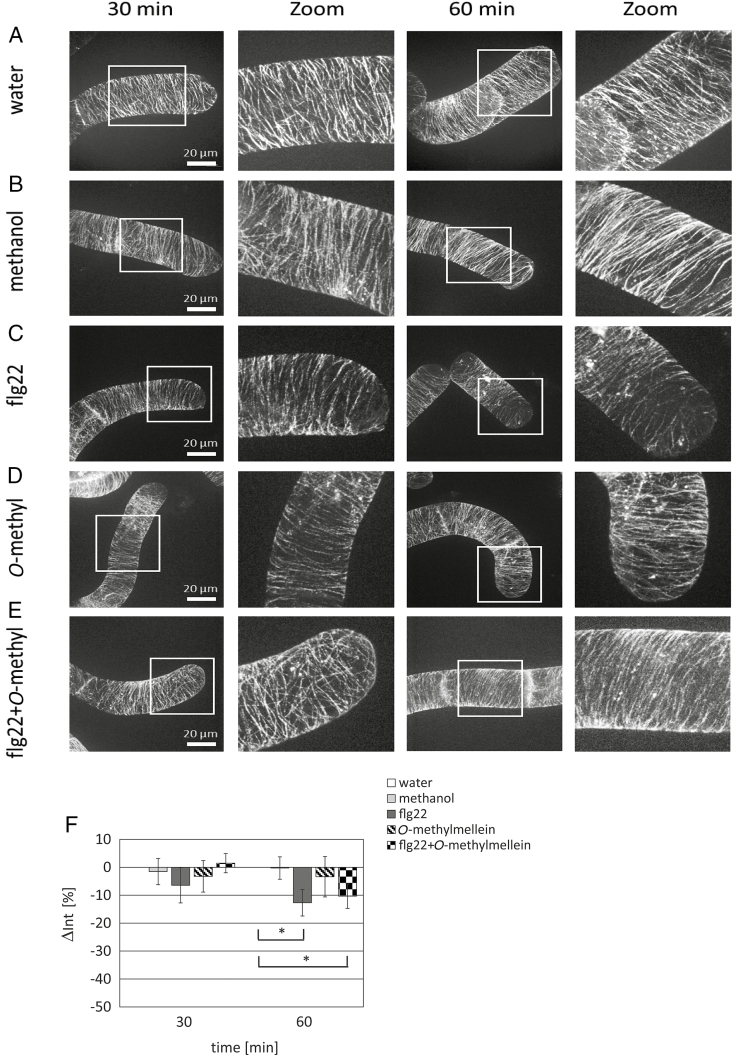
Microtubular responses to flg22 and *O*-methylmellein (*O*-methyl) in the cells of *V. rupestris* expressing the fluorescent tubulin marker GFP–*At*TUB6. The response of *Vitis* cells to the water control (A), 0.25% methanol solvent control (B), 1 µM flg22 (C), 25 µM *O*-methylmellein (D), or combined flg22 and *O*-methylmellein (E) for 30 and 60 min was observed by spinning-disc confocal microscopy. A representative confocal section from a z-stack along with different time points (30 and 60 min) and an enlargement of microtubules is shown. (F) Quantitative analysis of microtubule diameter in relative units at 30 and 60 min. Error bars indicate standard error of the mean; *P<0.05, **P<0.01 (Student’s *t*-test). Observations are representative of at least four independent experimental series with a population of 50 individual cells for each treatment. Scale bars: 20 μm.

Motivated by the previous result that the *E. lata* E16012 (BAF) extract, but not the *E. lata* E16012 (PDA) extract, induced microtubule depolymerization (Fig. 2), we wondered whether *O*-methylmellein would act on microtubules. We found that microtubules were partially depolymerized as compared with the water control (Fig. 7A), when the cells were incubated with flg22 for 30 or 60 min (Fig. 7C). However, *O*-methylmellein had no effects on microtubules (Fig. 7D), and even the combined treatment did not enhance the slight microtubule depolymerization seen for flg22 alone (Fig. 7E). When we quantified microtubule integrity over time, we found that only flg22 over 1 h was able to induce significant drops of microtubule integrity, no matter whether *O*-methylmellein was present or absent (Fig. 7F). Therefore, *O*-methylmellein cannot account for the microtubule-depolymerization activity of the BAF culture filtrate (Fig. 2).

**Fig. 8.**
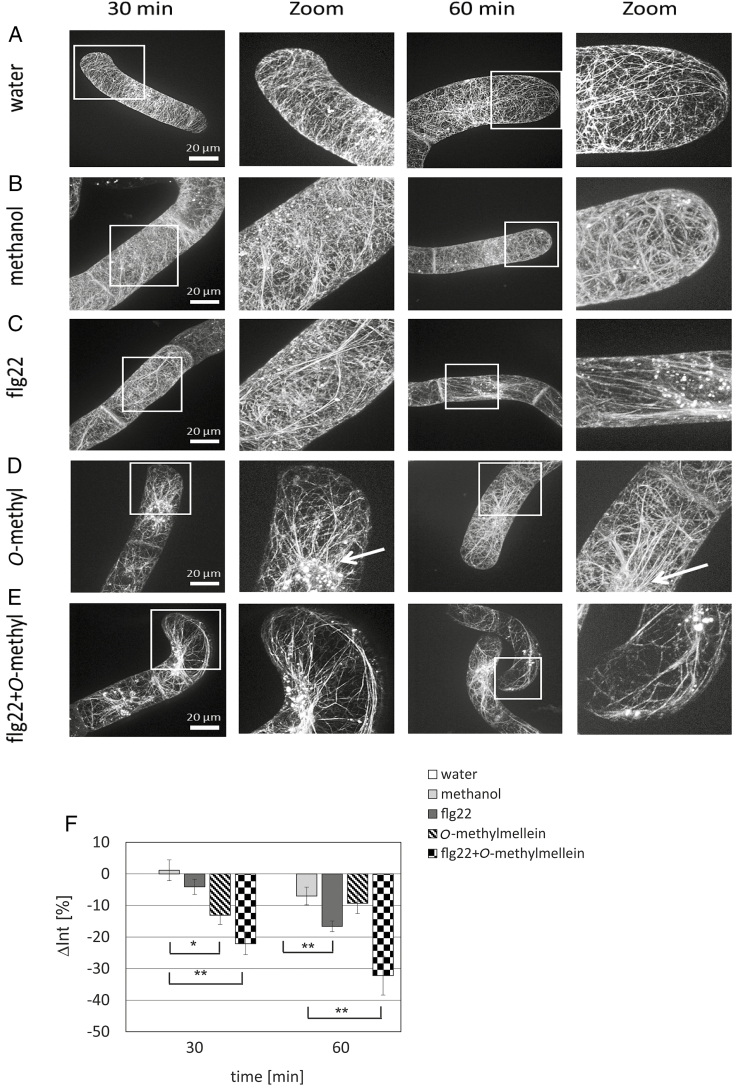
Responses of actin filaments to flg22 and *O*-methylmellein (*O*-methyl) in the cells of *V. vinifera* L. cv. ‘Chardonnay’ expressing the actin marker FABD2–GFP. The *V. vinifera* cells were incubated with the same treatments (A–E) as in the Fig. 7. (D) The cell nucleus in the treatment of *O*-methylmellein is marked by a white arrow. The GFP visualization and enlargement of actin filaments at different time points (30 and 60 min) is shown. (F) Quantification analysis of relative density of actin filaments at 30 and 60 min. Error bars indicate standard error of the mean; *P<0.05, **P<0.01 (Student’s *t*-test). Observations were representative of at least four independent experimental series with a population of 50 individual cells for each treatment. Scale bars: 20 μm.

At sites of attempted penetration of fungi and oomycetes, often radial actin bundles have been observed (Takemoto *et al.*, 2003; Opalski *et al.*, 2005). Moreover, several PAMPs and effectors were found to disrupt the actin cytoskeleton associated with the inhibition of plant defence (Park *et al.*, 2018). We, therefore, repeated the experiment with the same design, but this time following actin filaments. Both flg22 and *O*-methylmellein induced a depletion of these cortical strands, while longitudinal actin cables appeared (Fig. 8). This bundling was more pronounced for *O*-methylmellein as compared with flg22. For *O*-methylmellein, the bundled cables also contracted upon the nucleus (marked by an arrow in Fig. 8D). A quantification of actin depletion showed that the combined treatment was significantly more effective as compared with flg22 alone (Fig. 8F). This actin reorganization was strongly enhanced for the combined treatment (Fig. 8E). Here, already after 30 min, the cortical meshwork had disappeared almost completely, and the thick cables of actin had already strongly contracted to the nucleus. At 60 min, patches of actin appeared, characteristic of actin-nucleation sites and manifesting when actin is disassembled by actin-eliminating compounds (Maisch *et al.*, 2009).

### O-Methylmellein is not a fungal phytotoxin for grapevine cells

**Fig. 9.**
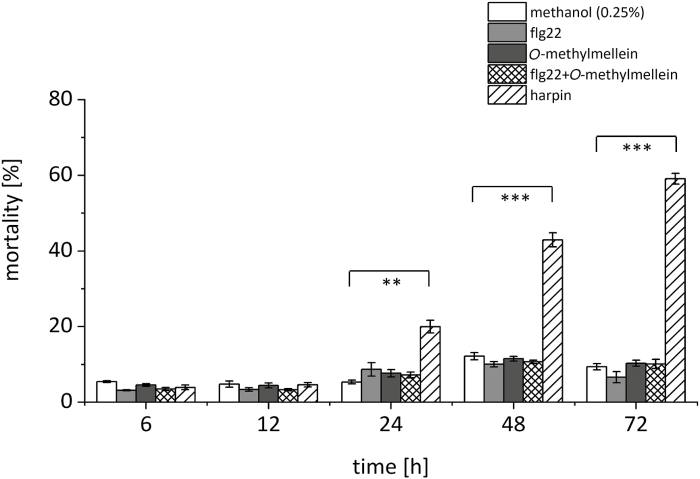
Effect of flg22 and *O*-methylmellein on cell mortality in the cell line *V. rupestris* GFP–*At*TuB6. Cells were incubated with 25 µM *O*-methylmellein, 1 µM flg22, or a combination of both compounds, along with 0.25% methanol as solvent control or 9 µg ml^−1^ harpin as a positive control for 6, 12, 24, 48, and 72 h. The cell mortality was scored for at least 1000 cells per sample. The ratio of the dead cells over the scored cells was calculated as the cell death frequency. Error bars indicate standard error of the mean; *P<0.05, **P<0.01, ***P<0.001 (Student’s *t*-test), *n*=3.

The outbreak of GTD symptoms has been discussed as being caused by the secretion of fungal phytotoxins. In fact, mellein and its derivatives have been reported to induce leaf necrosis in *Vitis vinifera* (Djoukeng *et al.*, 2009). The phytotoxic activity of *O*-methylmellein was measured by using *V. rupestris* TuB6 cells and an Evans Blue dye exclusion assay. *O*-Methylmellein could not activate any significant increase in mortality, even after prolonged treatment (Fig. 9). For comparison, the mortality induced by harpin in *V. rupestris* cells was found to already be 20% after 24 h, and increased to 60% after 72 h (Fig. 9). Thus, *O*-methylmellein is not acting as a phytotoxin, either alone or in combination with flg22.

## Discussion

Our rationale with regard to GTDs as novel type of disease is to focus on the signals exchanged between grapevine and pathogens. In order to detect those signals, we pursued a bioactivity-guided fractionation strategy using culture filtrates from *E. lata* leading to the identification of *O*-methylmellein as an amplifier of flg22-triggered basal immunity. This amplification was multiplicative, and correlated with a synergistic effect on actin filaments, without any significant effect upon microtubules.

These findings lead to the following questions that will be discussed below:

(i)   By which pathway can *O*-methylmellein stimulate flg22-triggered PTI?
(ii) What role does the cytoskeleton play in this pathway?
(iii) What might be the biological function of a fungal amplifier?

### Functional modularity of compounds in fungal culture extracts

Fungal culture filtrates have stimulated the appearance of typical hallmarks of grapevine immunity (Supplementary Table S3; Supplementary Fig. S2). However, the biological activities of those culture extracts varied depending on the strains and growth media. The finding that the same fungal isolate can produce different types of metabolites is not unusual and has been studied intensively for mycotoxins produced in food-contaminating fungi (Betina, 1989; Kokkonen *et al.*, 2005). Also for *E. lata*, metabolomic variability has been demonstrated (Mahoney *et al.*, 2005). To date, the factors that control these metabolic responses have remained elusive. This is also true for the current study—why *E. lata* strain E16012 generates significantly higher immunity activation in response to BAF medium as compared with PDA medium is not known. As to be expected, the functional difference extends beyond a single compound and is multi-faceted, as will be explained in the following.

The transcription of defence genes that encode enzymes of stilbene biosynthesis was induced by almost all the culture extracts (Supplementary Fig. S2). Since some stilbenes, such as resveratrol and oxidized stilbene oligomers, play a vital role in protecting grapevine from pathogen attacks, and can be induced by several PAMPs (Pezet *et al.*, 2004; Chang and Nick, 2012), we might conclude that some compounds present in those extracts act as PAMPs able to activate basal grapevine immunity.

To trim down complexity and verify the putative PAMPs, effectors, or ‘amplifiers’, we have therefore focused on the first step of phytoalexin synthesis, PAL, because this bioactivity was strongly amplified in the filtrate collected from *E. lata* strain E16012 upon cultivation in BAF over that seen for cultivation in PDA, such that there would be a realistic chance to arrive at a candidate molecule behind this differential activity. In fact, we succeeded in separating in the HPLC profile two regions that differentially activated *StSy* and *PAL* (Fig. 3), and in identifying *O*-methylmellein as a bona fide candidate correlated with the higher bioactivity (with respect to induction of *PAL*) in the *E. lata E16012* (BAF) culture filtrate.

The same conclusion has to be drawn for the reorganization of the microtubule network that was differentially triggered by the two culture filtrates (Fig. 2). This microtubule response correlated with the activation of phytoalexin-synthesis genes, and can be used as an early readout for a defence response in grapevine (Qiao *et al.*, 2010). The functional context of this microtubule response is not clear. While the microtubules have been proposed as tracks for the delivery of cell-wall components and also other defence agents to the fungal infection sites (Schmidt and Panstruga, 2011), this concept ignores that exocytosis in plants runs mostly through actin filaments (Boevink et al., 1998), and that depolymerization of cortical microtubules would impede, rather than promote, transport of such vesicles, because transport in cortical microtubule arrays requires sustained treadmilling (Shaw *et al.*, 2003).

### *O*-Methylmellein as an amplifier: what can we conclude on the pathway?

*O*-Methylmellein is a derivative of mellein, which was first isolated from the saprobiotic fungus *Aspergillus melleus* (Nishikawa, 1933). Our results have shown that *O*-methylemein is neither a candidate toxin that causes the foliar symptoms in GTDs (Fig. 9), nor an elicitor that activates grapevine basal immunity (Fig. 4A, B), but acts as a positive regulator on flg22-triggered defence gene transcription (Fig. 5). These findings indicate that *O*-methylmellein, a secondary metabolite of a wood-populating ascomycete (*E. lata*), acts as a weapon in interspecies competition by amplifying the basal immune response activated by bacterial elicitor flg22. Thus, the interaction of flg22 and *O*-methylmellein is multiplicative, and not additive, which means that the signalling triggered by *O*-methylmellein and that triggered by flg22 must be shared to a certain extent. From the measurement of extracellular pH, we know that the point of merger must be downstream of the calcium channel.

Consistent with this supposition, the calcium influx blocker GdCl_3_ inhibited the amplification of flg22-triggered gene expression by *O*-methylmellein only slightly. Instead, a significant (albeit not complete) inhibition was observed when the membrane-located NADPH oxidase RboH was blocked by DPI (Fig. 6). In grapevine cells, RboH participates in defence-related cell death in response to the bacterial elicitor harpin (Chang and Nick, 2012), or the product of plant oxylipin metabolism, 3-*cis*-hexenal (Akaberi *et al.*, 2018), and was also found to be necessary for the resulting gene activation (Chang *et al.*, 2011). Thus, *O*-methylmellein as an amplifier shares a specific signature (dependence on RboH, independence from calcium influx) with cell-death-related defence, while not culminating in cell death. Thus, there must be a point where *O*-methylmellein signalling diverges from the signalling leading to programmed cell death.

There is a further, very specific, hallmark of defence-related cell death: massive and rapid remodelling of actin filaments that has been found for the response to both harpin (Chang *et al.*, 2015) and 3-*cis*-hexenal (Akaberi *et al.*, 2018). We checked this phenomenon and found that *O*-methylmellein induced actin remodelling, and more importantly, boosted actin remodelling in response to flg22 in a synergistic manner (Fig. 8). This effect was specific, because it was not seen for the microtubule network (Fig. 7).

Thus, supported by (non-intuitive) implications that have been experimentally confirmed, the following model emerges for the signalling events triggered by *O*-methylmellein: activation of RboH, leading to an apoplastic oxidative burst, penetration of reactive oxygen species (Eggenberger *et al.*, 2017), probably through aquaporins, into the cytoplasm, remodelling of actin (Chang *et al.*, 2015), and activation of phytoalexin genes. The divergence from the signalling leading to defence-related cell death seems to be close to actin remodelling. The convergence with the signalling deployed by flg22 is downstream of calcium influx, but upstream of gene expression. A straightforward hypothesis would locate this convergence at mitogen-activated protein kinase signalling. A testable implication of this hypothesis would be that treatment with the specific inhibitor PD98059 should disrupt the amplifier activity of *O*-methylmellein (Chang and Nick, 2012).

While the individual events of this pathway have been demonstrated, the model sketched above does not answer the question of why *O*-methylmellein can deploy this signalling only in the presence of flg22, and not by itself. What is the reason that this compound cannot act as an elicitor, but only as an (multiplicative) amplifier? In the following, we will discuss this as a possible consequence of the cytoskeletal activity.

### Does actin play a role in elicitor sensitivity?

The remodelling of actin filaments is clearly detectable already 30 min after addition of *O*-methylmellein (Fig. 8) and thus clearly precedes the amplification of flg22-induced transcripts (Fig. 5). How might actin remodelling interfere with defence? Most work on this topic has focused on so-called penetration resistance, in which actin filaments reorganize around a site of attempted penetration and are involved in the deposition of callose, as well as in the accumulation of vesicles with phytoalexins or defence proteins (reviewed in Day *et al.*, 2011). A further phenomenon that has attracted attention is the movement of the nucleus towards the penetration site (Gus-Mayer *et al.*, 1998). The functional relevance of this phenomenon is not clear (for review see Griffis *et al.*, 2014); it might be rather a side phenomenon and caused by the rapid accumulation of actin filaments around the nucleus, because the perinuclear actin cage tethers the nucleus in concert with the plant-specific class XIV kinesin KCH1 (Frey *et al.*, 2010; Durst *et al.*, 2014).

Although these reports show the importance of actin, they do not really help in understanding how the remodelling of actin should lead to an amplifying effect of flg22-triggered gene expression. A possible mechanism might be the endocytotic uptake of the cognate flg22 receptor, FLS2 (Robatzek *et al.*, 2006), a process that is dependent on actin.

Thus, the amplifier effect of *O*-methylmellein can be explained by a working model in which reactive oxygen species-dependent actin remodelling increases the sensitivity towards flg22, by prolonging the lifetime of the flagellin receptor at the membrane.

### Outlook: hunting the function of the modifier

The concept of a fungal signal that boosts the defence response of the host appears counterintuitive, at first sight. However, a second look makes clear that amplifiers can confer a selective advantage under the conditions of colonization of grapevine trunks by microbial consortia, which include both fungi and bacteria (Bruez *et al.*, 2015; Gramaje *et al.*, 2018). In the world of microbes, competition for limited resources is commonly decided by chemical warfare, for instance by secretion of anti-microbial compounds that will weaken the competitor, but leave the donor unharmed because it harbours a mechanism to degrade the compound. In fact, *O*-methylmellein has been found to exert high fungal toxic activity against the GTD-associated fungus *Botryosphaeria obtusa* as a potential competitor, and *Botrytis cinerea*, a necrotrophic generalist (Glauser *et al.*, 2009). However, the competition might run indirectly through a ‘rail shot’ by leaving the job of killing the competitor to the host. This would allow *E. lata* to outcompete bacteria. If *O*-methylmellein acted as ‘borrowed knife’ (Sun, 1991), it will be necessary to understand, how *E. lata* itself can escape the immune response of the host. A third possibility should be kept in mind, however. The dominant gene activation seen in response to the amplifier effect of *O*-methylmellein concerns *PAL*, the first committed step of the phenylpropanoid pathway. This pathway generates not only phytoalexins of the host, but also monolignols, i.e. the building blocks for the main food source of the fungus. The fact that the chemical factors leading to stimulation of *PAL* can be separated from those responsible for the stimulation of *StSy* (Fig. 3A) would fit a scenario in which the fungus reprogrammes host metabolism towards lignin production, while simultaneously suppressing (by different signals) the phytoalexin-generating side branch of this pathway.

No matter which of these three scenarios is used to describe the interaction with host and competitors, all three possibilities would require metabolic reprogramming of *E. lata*, which on the molecular level should become visible as activation of otherwise silent genes, such as the members of the extensive polyketide synthase clusters. The (circumstantial) discovery that these silent potencies can be stimulated by cultivation in BAF medium indicates that the metabolic reprogramming is regulated by external factors. These might be signals from the host plant itself, or signals from competing microorganisms. To determine the real biological function of *O*-methylmellein will require pinning down the context under which it is generated. We have, therefore, launched a project in which we will co-cultivate different fungal signal ‘donors’ with grapevine cells as signal ‘receptors’ in a manner in which they can chemically interact but remain physically separate.

## Supplementary data

Supplementary data are available at *JXB* online.

Fig. S1. MS information for *O*-methylmellein, siccayne, and eutypinol in *Eutypa lata* IBWF E16012 (BAF) extract.

Fig. S2. Phytoalexin-synthesis genes expression with different culture extract treatments.

Fig. S3. Dose response of apoplastic alkalinization to *O*-methylmelleim over time.

Table S1. HPLC-MS analysis results of compositions of *Eutypa lata* IBWF E16012 (BAF) filtrate and E16012 (PDA) filtrate.

Table S2. List of oligonucleotide primers used for expression analysis by semi-quantitative and quantitative PCR.

Table S3. Extracellular alkalinization of *V. rupestris* cells in the presence of fungal culture filtrates.

eraa152_suppl_Supplementary_Figures_S1-S3-and-Tables_S1-S3

## Data availability

Data are available upon request to the corresponding author.

## Abbreviations

DPI: diphenylene-iodonium chloride
ETI: effector-triggered immunity
FLS2: flagellin-sensing 2
GFP: green fluorescent protein
GTD: grapevine trunk disease
PAMP: pathogen-associated molecular pattern
PDA: potato dextrose agar
PTI: PAMP-triggered immunity
RboH: respiratory burst oxidase homologue
