# Supplementary material for: Hunting modulators of plant defence: the grapevine trunk disease fungus Eutypa lata secretes an amplifier for plant basal immunity
Source: J Exp Bot. 2020 Mar 3;71(12):3710–24. doi: 10.1093/jxb/eraa152 (PMC7475250; doi:10.1093/jxb/eraa152)
Supplement: eraa152_suppl_Supplementary_Figures_S1-S3-and-Tables_S1-S3 [file eraa152_suppl_supplementary_figures_s1-s3-and-tables_s1-s3.pdf]

Supplementary Data

Title

Hunting modulators of plant defence - the Grapevine Trunk Disease fungus *Eutypa lata* secretes an amplifier for plant basal immunity

Authors

Pingyin Guan[1,*], Terigele[1], Florian Schmidt[2], Michael Riemann[1], Jochen Fischer[2], Eckhard Thines[2], Peter Nick[1]

[1]Molecular Cell Biology, Botanical Institute, Karlsruhe Institute of Technology, Fritz-Haber-Weg 4, 76131 Karlsruhe, Germany

[2]Institut für Biotechnologie und Wirkstoff-Forschung gGmbH, Erwin-Schrödinger-Str. 56, 67663 Kaiserslautern

*Author to whom correspondence should be addressed:

Pingyin Guan, Molecular Cell Biology, Botanical Institute, Karlsruhe Institute of Technology, Fritz-Haber-Weg 4, 76131 Karlsruhe, Germany.

Tel: +49 721 608 42142, Fax: +49 721 608 44193, Email: pingyin.guan@kit.edu

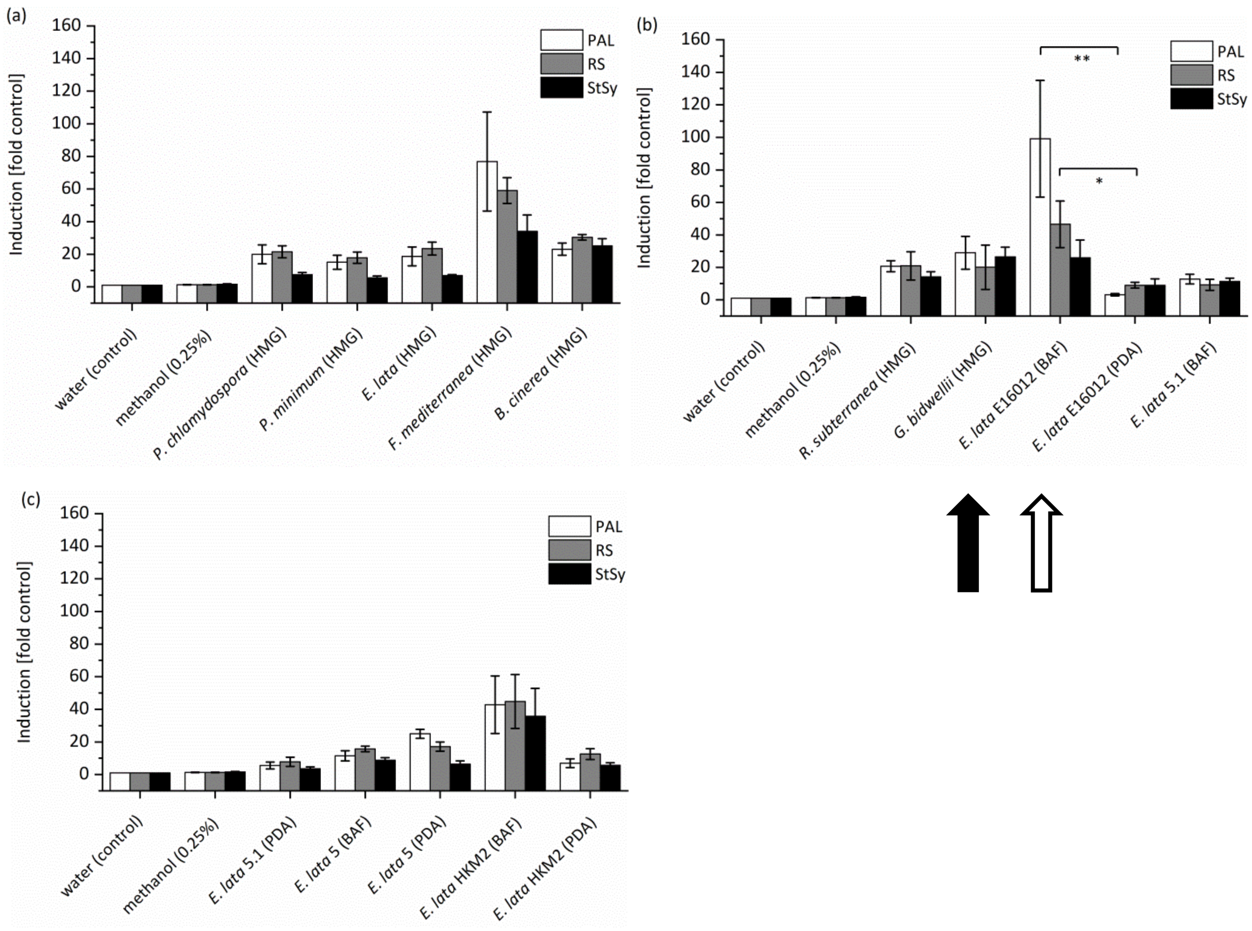

Fig. S1. Phytoalexin-synthesis genes expression to different cultural extract treatments. The treatment concentration of fungal cultural filtrates [(a) *Phaeomoniella chlamydospora* (HMG), *Phaeoacremonium minimum* (HMG), *Eutypa lata* (HMG), *Fomitiporia mediterranea* (HMG), *Botrytis cinerea* (HMG); (b) *Roesleria subterranea* (HMG), *Guignardia bidwellii* (HMG), *Eutypa lata* IBWF E16012 (BAF) (black arrow), *Eutypa lata* IBWF E16012 (PDA) (white arrow), *Eutypa lata* 5.1 (BAF); (c) *Eutypa lata* 5.1 (PDA), *Eutypa lata* 5 (BAF), *Eutypa lata* 5 (PDA), *Eutypa lata* HKM2 (BAF), *Eutypa lata* HKM2 (PDA)] was 25 μg ml^{-1} cultural filtrates, 0.25% methanol acted as the solvent control and water was used as the control. The transcription of genes *PAL*, *RS*, *StSy* was measured by qPCR using elongation factor 1α as an internal standard. Error bars stand ± standard error (SE) of the mean; *, $P < 0.05$ (Student's t-test), n=3.

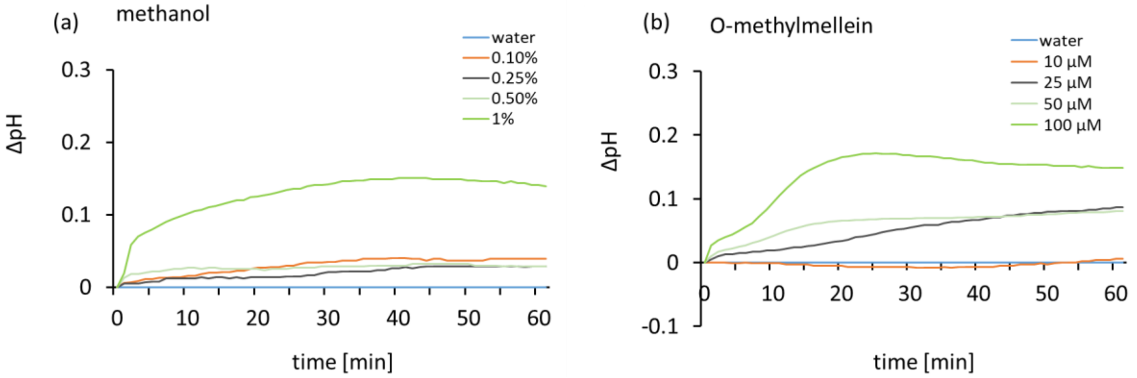

Fig. S2. Dose response of apoplastic alkalinisation to O-methylmelleim over time. Extracellular pH was measured in response to 0.1% - 1% methanol (a) and 10 μM – 100 μM O-methylmellein (b) along with time for 60 min. Water as the solvent control. The experiments were repeated at least five times.

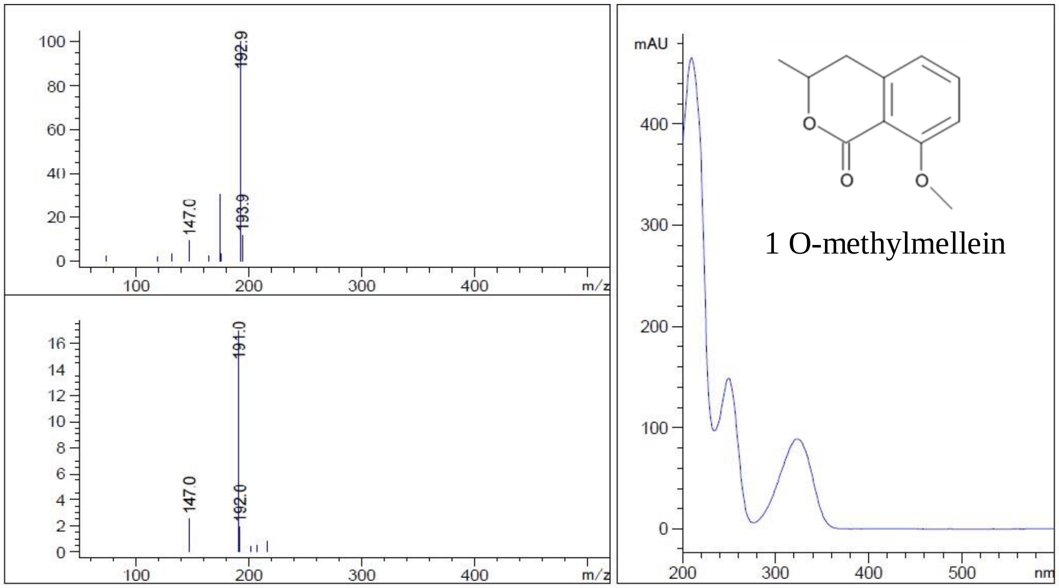
100
80
60
40
20
0
192.9
147.0
193.9
100
200
300
400
m/z
16
14
12
10
8
6
4
2
0
191.0
147.0
192.0
mAU
400
300
200
100
0
200
300
400
500
nm
O
O
O
1 O-methylmellein

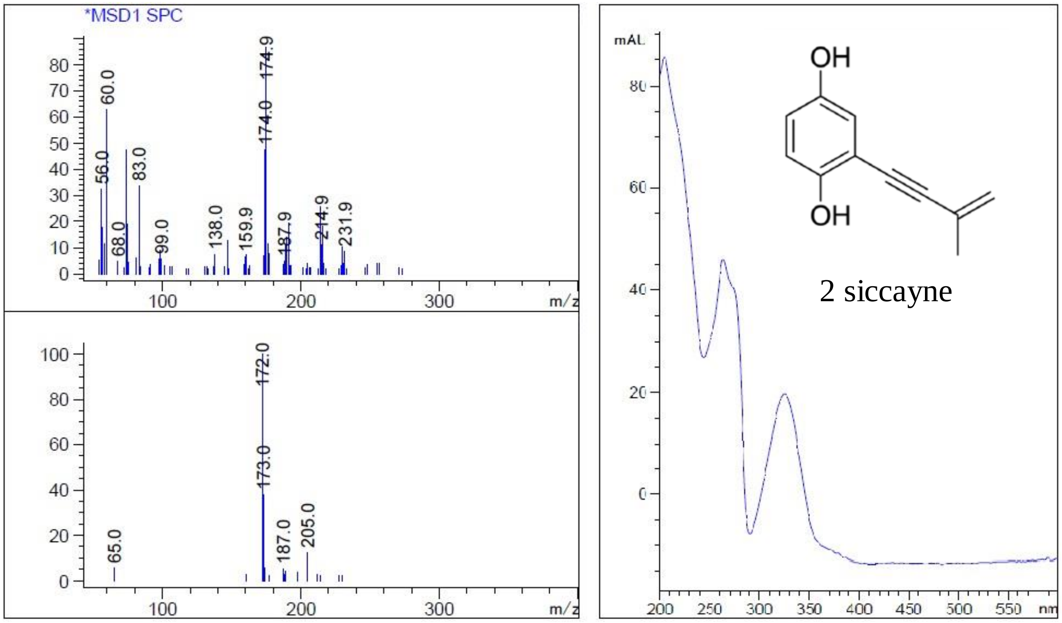
*MSD1 SPC
80
70
60
50
40
30
20
10
0
60.0
56.0
68.0
83.0
99.0
138.0
159.9
174.9
174.0
187.9
214.9
231.9
100
200
300
m/z
100
80
60
40
20
0
65.0
172.0
173.0
187.0
205.0
mAU
80
60
40
20
0
200
250
300
350
400
450
500
550
nm
OH
OH
2 siccayne

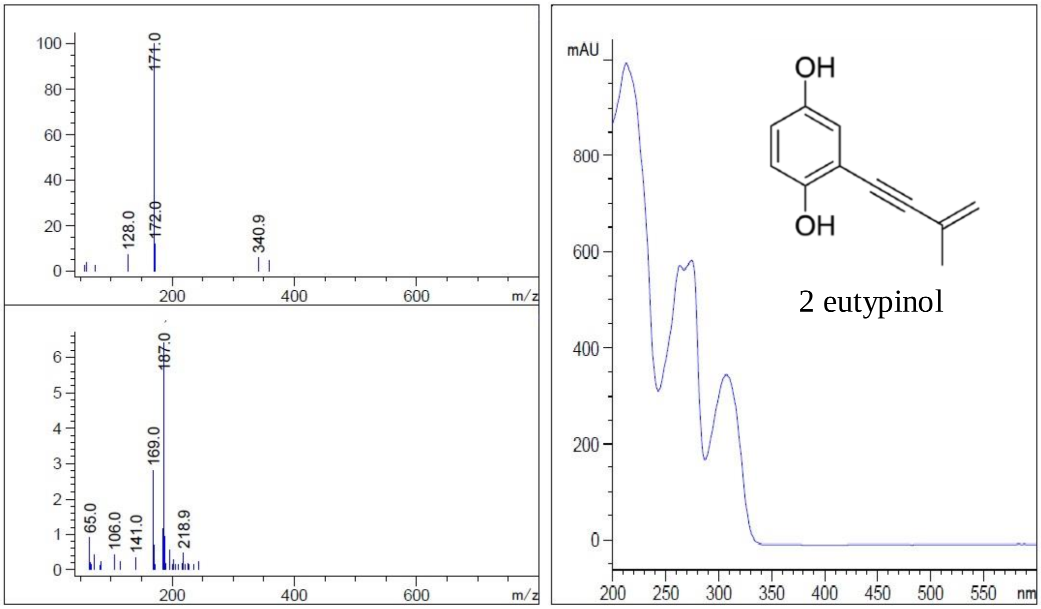

Fig. S3. The MS information of (1) O-methylmellein, (2) siccayne and eutypinol in *Eutypa lata* IBWF E16012 (BAF) extract.

Table S1. List of oligonucleotide primers used for expression analysis by semi-quantitative and quantitative PCR.

EF1α, elongation factor 1α; PAL, phenylalanine ammonia lyase; RS, resveratrol synthase; StSy, stilbene synthase; MC2, metacaspase 2； MC5, metacaspase 5； JAR1, jasmonate-resistant 1; JAZ1, the jasmonate ZIM/tify-domain protein 1, a transcriptional repressor.

**Name**	**GenBank accession no.**	**Primer sequence 5'-3'**	**Reference**
EF1α	EC959059	Sense: 5'-GAACTGGGTGCTTGATAGGC-3'<br>Antisense: 5’-AACCAAAATATCCGGAGTAAAAGA-3’	Reid *et al.* (2006)
PAL	X75967	Sense: 5’-TGCTGACTGGTGAAAAGGTG-3’<br>Antisense: 5’-CGTTCCAAGCACTGAGACAA-3’	Belhadj *et al.* (2008)
RS	AF274281	Sense: 5'-TGGAAGCAACTAGGCATGTG-3'<br>Antisense: 5'-GTGGCTTTTTCCCCCTTTAG-3'	Duan *et al.* (2015)
StSy	X76892	Sense: 5'-CCCAATGTGCCCACTTTAAT-3’<br>Antisense: 5'-CTGGGTGAGCAATCCAAAAT-3'	Duan *et al.* (2015)
VvMC 2	KC494645	Sense: 5'-TGGGGAGGTCATTTCCTTTAG-3'<br>Antisense: 5'-GGTTGATCGCATTGAATTTAGC-3’	Gong *et al.* (2019)
VvMC 5	KC494648	Sense: 5'-GAGGGTTGCCGCATTACGA-3’<br>Antisense: 5’-GCACCTTGCACGGTTTGGT-3’	Gong *et al.* (2019)
JAR1	XM_002280702.2	Sense: 5’-GAGAATTGCGGATGGTGATA-3’<br>Antisense: 5’-CTAAAGGCGAAAGAGGTT-3’	Figueiredo *et al.* (2015)
JAZ1	JF900329	Sense: 5’-TGCAGTCTGTTGAGCCAATACATA-3’<br>Antisense: 5’-CACGTTTCCGGACTTCTTTACAC-3’	Ismail A *et al.* (2012)

Table S2. Extracellular alkalinisation of *V. rupestris* cells in the presence of fungal culture filtrates.

The ΔpH was recorded after 20 min of suspension cells incubated with 25 μg ml^{-1} culture filtrates, 0.25% methanol as the solvent control, water as the control. The experiments were repeated at least five times.

**Medium**	**Organismus**	**ΔpH (after 20 min)**
water	--	0
methanol	--	-0.0055
HMG	*Phaeomoniella chlamydospora*	0.60
HMG	*Phaeoacremonium aleophilum*	0.54
HMG	*Eutypa lata*	0.55
HMG	*Fomitiporia mediterranea*	0.21
HMG	*Botrytis cinerea*	0.24
HMG	*Roesleria subtereanea*	0.13
MEM	*Guignardia bidwellii*	0.24
BAF	*Eutypa lata* E16012	0.40
PDA	*Eutypa lata* E16012	0.39
BAF	*Eutypa lata* 5.1	0.22
PDA	*Eutypa lata* 5.1	0.24
BAF	*Eutypa lata* 5	0.01
PDA	*Eutypa lata* 5	0.05
BAF	*Eutypa lata* HKM2	0.14
PDA	*Eutypa lata* HKM2	0.22

Table S3. HPLC-MS analysis results of compositions of *Eutypa lata* IBWF E16012 (BAF) (a) filtrate and E16012 (PDA) (b) filtrate.

(a) BAF medium					
**Well-position**	**Compound**	**Notes**	**Well-position**	**Compound**	**Notes**
A3 (0.51 min)	Unidentified	-	A6 (1.33 min)	Unidentified	UV spectra similar to B1
A10 (2.51 min)	Unidentified	-	B9 (3.77 min)	Unidentified	-
B5 (4.79 min)	Unidentified	UV spectra similar to B3 and C3	B3 (5.29 min)	Unidentified	UV spectra similar to B5 and C5
B1 (5.98 min)	Unidentified	UV spectra similar to A6	C2 (6.26 min)	Unidentified	-
C3 (6.69 min)	Unidentified	UV spectra similar to B3 and C5	C7 (7.61 min)	Unidentified	-
C6 (7.35 min)	Unidentified	-	C9 (8.07 min)	FS E16012-4	UV spectra and mass match
C12 (8.85 min)	Unidentified	-	D11 (9.31 min)	O-methylmellein	UV spectra and mass match
D9 (9.92 min)	Siccayne/ Eutypinol	UV spectra match; mass not available	D3 (11.31 min)	Unidentified	-
D2 (11.68 min)	Unidentified	-	E1 (12.11 min)	Unidentified	-
E10 (14.50 min)	Unidentified	-	E3 (12.57 min)	Unidentified	-
E6 (13.40 min)	Unidentified	UV spectra similar to E7, F11 and F12	E7 (13.67 min)	Unidentified	UV spectra similar to E6, F11 and F12
F12 (15.10 min)	Unidentified	UV spectra similar to E6, E7 and F11	F11 (15.30 min)	Unidentified	UV spectra similar to E6, E7 and F12

(b) PDA medium					
**Well-position**	**Compounds**	**Notes**	**Well-position**	**Compounds**	**Notes**
A6 (1.35 min)	Unidentified	-	B10 (3.61 min)	Unidentified	-
B8 (4.05 min)	Unidentified	-	B6 (4.57 min)	Unidentified	-
B5 (4.95 min)	Unidentified	-	B1 (5.72 min)	Unidentified	-
C1 (6.20 min)	Unidentified	-	C2 (6.46 min)	Unidentified	-
C4 (6.83 min)	Unidentified	-	C6 (7.30 min)	Unidentified	-
C9 (8.10 min)	FS E16123-1	UV spectra and mass match	D12 (9.05 min)	O-methylmellein	UV spectra and mass match
D10 (9.61 min)	Siccayne/ Eutypinol	UV spectra match; mass not available	D6 (10.73 min)	Unidentified	-
D4 (11.03 min)	Unidentified	-	D2 (11.60 min)	Unidentified	-
D1 (11.90 min)	FS E16123-5	UV spectra and mass match	E2 (12.33 min)	FS E16123-3	UV spectra and mass match
E5 (13.10 min)	Unidentified	-	E9 (14.10 min)	Unidentified	-

References

Belhadj, A., N. Telef, C. Saigne, S. Cluzet, F. Barrieu, S. Hamdi, and J.-M. Mérillon. 2008. Effect of methyl jasmonate in combination with carbohydrates on gene expression of PR proteins, stilbene and anthocyanin accumulation in grapevine cell cultures. *Plant Physiology and Biochemistry* **46,** 493-499.

Duan, D., D. Halter, R. Baltenweck, C. Tisch, V. Troster, A. Kortekamp, P. Hugueney, and P. Nick. 2015. Genetic diversity of stilbene metabolism in *Vitis sylvestris*. *Journal of Experimental Botany* **66,** 3243-3257.

Figueiredo, A., F. Monteiro, and M. Sebastiana. 2015. First clues on a jasmonic acid role in grapevine resistance against the biotrophic fungus *Plasmopara viticola*. *European Journal of Plant Pathology* **142,** 645-652.

Gong, P., M. Riemann, D. Dong, N. Stoeffler, B. Gross, A. Markel, and P. Nick. 2019. Two grapevine metacaspase genes mediate ETI-like cell death in grapevine defence against infection of *Plasmopara viticola*. *Protoplasma* **256,** 1-19.

Ismail, A., M. Riemann, and P. Nick. 2012. The jasmonate pathway mediates salt tolerance in grapevines. *Journal of Experimental Botany* **63,** 2127-2139.

Reid, K.E., N. Olsson, J. Schlosser, F. Peng, and S.T. Lund. 2006. An optimized grapevine RNA isolation procedure and statistical determination of reference genes for real-time RT-PCR during berry development. *BMC Plant Biology* **6,** 27-37.
